# Integrative investigation of the TF–miRNA coregulatory network involved in the inhibition of breast cancer cell proliferation by resveratrol

**DOI:** 10.1002/2211-5463.13344

**Published:** 2021-12-12

**Authors:** Yongfeng Zhou, Jing Zhang, Wei Li, Daoyu Zhang, Zhengzhu Wang, Yanhui Zhai, Hao Yu, Ziyi Li

**Affiliations:** ^1^ Key Laboratory of Organ Regeneration and Transplantation of Ministry of Education First Hospital Jilin University Changchun China; ^2^ College of Animal Sciences Jilin University Changchun China

**Keywords:** integrated analysis, MCF‐7, microRNA, resveratrol, transcription factor

## Abstract

Resveratrol is a polyphenol with antiaging and anticancer effects. Most previous studies used a single analysis to determine the key functions of resveratrol in inhibiting cancer progression. However, most of the signal transmission pathways in biological processes are multilevel. We used bioinformatics to elucidate the mechanism of resveratrol inhibition of breast cancer development. The mRNA expression profile of GSE25412 from the National Center for Biotechnology Information (NCBI) and the microRNA (miRNA) expression profile of PubMed identifier (PMID) 26890143 were integrated. *De novo* motifs were used to obtain predicted transcription factor (TF) motifs for differentially expressed genes. The regulatory effect of resveratrol on key nodes in the comprehensive analysis results was verified. The TF–miRNA–mRNA interaction network based on the STITCH and miRDB databases showed that resveratrol exerted a dual inhibitory effect by activating inhibitory TFs to block the cell cycle and inhibit miRNAs from upregulating apoptosis. However, these two processes did not work completely independently. *TP53* is the dominant hub gene associated with the cell cycle and apoptosis throughout the TF–miRNA network. Kaplan–Meier plotter analysis found that resveratrol‐induced expression changes in key RNAs, such as *E2F2*, *JUN*, *FOS*, *BRCA1*, *CDK1*, *CDKN1A*, *TNF,* and hsa‐miR‐34a‐5p, significantly improved the prognosis of breast cancer patients, which was further verified using real‐time quantitative PCR (qPCR) and western blotting. This study constructed a TF–miRNA regulatory network with *TP53* and *E2F* as the main central genes to elucidate the molecular mechanism of resveratrol in the treatment of breast cancer.

## Graphical abstract

Resveratrol is a polyphenol with anti‐aging and anti‐cancer effects, which can significantly inhibit cell proliferation in MCF‐7 cells. Here, we found that resveratrol induces cell cycle arrest and promotes apoptosis by regulating transcription factors and microRNA networks. In addition, TP53 and E2F2 are hub proteins in the regulation network.

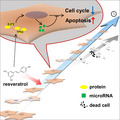

**Abbreviations**
ANOVA: one‐way analysis of variance
ATM: ATM serine/threonine kinase
ATR: ATR serine/threonine kinase
BAX: BCL2 associated X, apoptosis regulator
BCL2: BCL2 apoptosis regulator
BRCA1: BRCA1 DNA repair associated
CCK‐8: cell counting kit‐8
CDK1: cyclin dependent kinase 1
CDKN1A: cyclin dependent kinase inhibitor 1A
DAVID: database for annotation, visualization and integrated discovery
DEG: differentially expressed gene
DRAM: DNA damage‐regulated autophagy modulator 1
E2F2: E2F transcription factor 2
ER: estrogen receptor
FOS: Fos proto‐oncogene, AP‐1 transcription factor subunit
GADD45A: growth arrest and DNA damage inducible alpha
GAPDH: glyceraldehyde‐3‐phosphate dehydrogenase
GEO: gene expression omnibus
GO: gene ontology
HER2/neu: human epidermal growth factor receptor‐2
JUN: Jun proto‐oncogene, AP‐1 transcription factor subunit
miRNA: microRNA
NCBI: National Center for Biotechnology Information
OD: optical density
PARP: poly(ADP‐ribose) polymerase 1
PMID: PubMed identifier
PR: progesterone receptor
SDS/PAGE: sodium dodecyl sulfate/polyacrylamide gel electrophoresis
TF: transcription factor
TNF: tumor necrosis factor
TNFRSF21: TNF receptor superfamily member 21
TNFRSF9: TNF receptor superfamily member 9
TNFSF10: TNF superfamily member 10
TP53: tumor protein p53
TP53INP1: tumor protein p53 inducible nuclear protein 1
TRIAP1: TP53 regulated inhibitor of apoptosis 1

Breast cancer is the most diagnosed female cancer worldwide, and it is a perplexing puzzle due to its high heterogeneity and complexity [1]. Breast cancer is also the most common type of cancer in women in developing countries, and its incidence has increased significantly over the past two decades [2]. Approximately 304,000 new breast cancer cases are diagnosed annually, which makes it the leading cause of cancer in women in South‐East Asia, and approximately 70,000 women die from the disease in China alone [3]. The treatment of breast cancer is a great challenge because it is a heterogeneous disease involving different subtypes with different but specific characteristics. Breast cancer is classified by stage, histology, and grade differentiation, and the expression of estrogen receptor (ER), progesterone receptor (PR), and human epidermal growth factor receptor‐2 (HER2/neu) determines the response of breast cancer cells to currently available chemotherapeutic drugs. Many chemotherapeutic agents that are used to treat cancer are derived from plants, especially fruits, leaves, and flowers. The public currently prefers herbal medicine to synthetic medicine because herbal medicine contains natural active compounds that support human health.

Resveratrol is a naturally occurring polyphenol found in plants, such as grapes, blueberries, mulberries, and other plants. Cottart et al. showed that resveratrol was well tolerated by the human body [4]. Extensive scientific research shows that resveratrol has a variety of therapeutic effects, including antioxidant, antibacterial, cardioprotective, and antitumor effects [5]. Resveratrol mediates apoptosis and induces cell cycle arrest to exert its anticancer effects [6, 7, 8]. The effects of resveratrol on breast cancer are controversial. Our previous research on other cell lines suggested positive and negative effects, but positive results predominated [9].

MicroRNAs (miRNAs) are a class of small and noncoding RNA molecules that regulate gene expression after transcription. Recent studies showed that miRNAs played an important role in regulating tumor proliferation, differentiation, and apoptosis [10, 11]. Numerous studies confirmed that the targets of miRNAs were closely related to tumor progression in breast cancer [12]. Some miRNAs have antiapoptotic effects, and other miRNAs promote apoptosis. Common regulators of genes include transcription factors (TFs), which are primarily responsible for pretranscriptional regulation. Information from networks of miRNAs, TFs, and mRNAs may be used to identify cancer subtypes. This information provides novel insights into the mechanisms regulating each cancer subtype.

The rapid accumulation of large amounts of ‘‐omic’ data, especially transcriptomic data, provides an opportunity to better understand the underlying mechanisms of cancer development and metastasis. We constructed a TF‐miRNA coregulatory network from the integration of gene modules and their TFs and miRNA regulators from the public dataset provided in the Gene Expression Omnibus (GEO). Secondary mining of information and experimental validation helped reveal the potential therapeutic mechanisms of resveratrol in breast cancer.

## Materials and methods

### Gene expression profile data

Microarray data on gene expression (GSE25412) were downloaded from GEO. The included dataset met the following criteria. (a) It used a human breast adenocarcinoma‐derived MCF‐7 cell line. (b) It contained vehicle‐control groups. (c) It contained 3 samples for each group. A large quantity of data can reliably reveal differentially expressed genes (DEGs), but Ismael Leon‐Galicia et al. only analyzed data on genetic damage repair [14]. We further described the effect of resveratrol on MCF‐7 cells in a more comprehensive manner. To determine the effects of resveratrol on miRNA, we referred to Venkatadri et al. and the results of PCR array of miRNA expression in cells treated with resveratrol. The miRNA was screened using a threshold of 2‐fold change in the expression level. The selected miRNAs were used for further analysis and network construction (Venkatadri R, Muni T, et al., 2016; PMID: 26890143; Copyright and License information: http://creativecommons.org/licenses/by/4.0/) [13].

### Analysis of datasets

The limma software package [14] in r software was used to normalize and base‐logarithmically transform the matrix data for the GEO dataset. DEGs were defined as |log_2_FC| > 2 and FDR < 0.05. To more realistically show the macroscopic effect of resveratrol on gene expression function, we used the parameters of FDR < 0.1 and |log_2_FC| > 1 to draw a clustering heatmap using K‐Means. The Database for Annotation, Visualization and Integrated Discovery (DAVID) [15] was used for functional enrichment analysis. Each cluster was subjected to enrichment analysis using Gene Ontology (GO) annotation to reveal the possible functional classification.

The gradual improvement of the motif database of transcription factors allowed us to estimate the transcription status of DEGs. Motif discovery and enrichment were evaluated using Homer (v4.11) [16] (findMotifs.pl –start 2000 –end 2000). *De novo* motifs with a ‘best match score’ > 0.60 were ranked based on enrichment (−log_10_p_value) and plotted in the R package ggplot2. We estimated the target protein of resveratrol using the STITCH database [17].

The targeting relationship of miRNAs was obtained from the miRTarBase [18] and miRNet [19] databases, and a network side list was established. First, we isolated miRNA target genes that differed by more than 2 times from the list and performed KEGG pathway enrichment. Second, we combined the list of differentially expressed genes to obtain a possible miRNA‐mRNA regulatory network based on resveratrol treatment. The network was constructed and analyzed in Cytoscape, and transcription factors were labeled with reference to the TRRUST [20] database.

To better show the regulatory relationship between miRNAs and mRNAs, we imported the annotated list of differential genes into Origin 2021 and RCircos to generate a sunburst and Circos diagram. Sunburst diagram construction used the control and 150 mm groups of quantitative data, where the classification information was established by KEGG pathway and part of the manual. A Circos diagram was constructed on the UCSC hg19 genome annotation by the RCircos package.

**Fig. 1:**
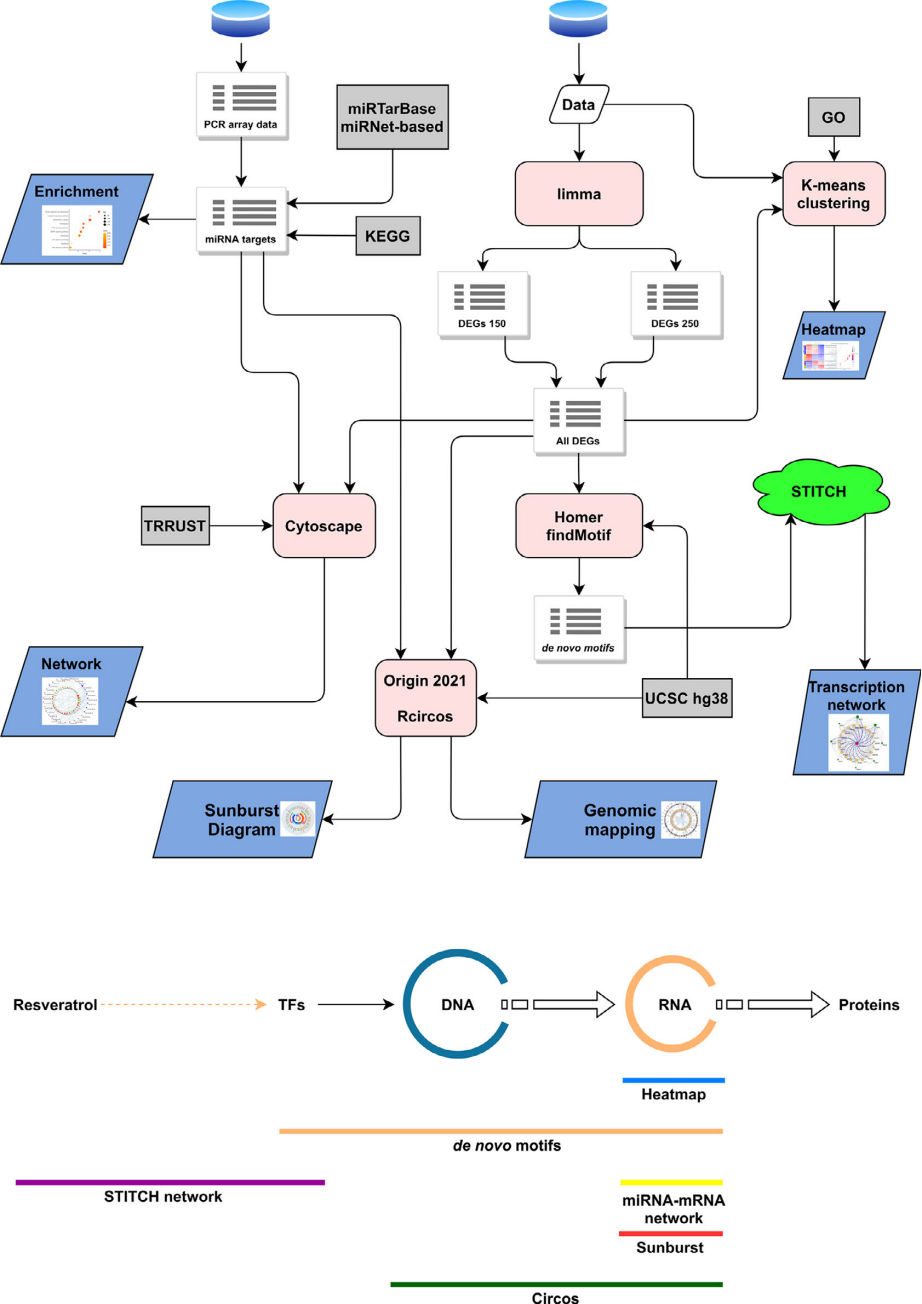
Overview of the data analysis process of the database source. The top half of the graph showed the data analysis process. The blue cylinder represented the public database; the gray rectangle represented the reference database; the pink rounded rectangle indicated the software; green clouds indicated online tools; and the blue parallelogram indicated the output results. The lower half of the graph represented the mapping of the data analysis results to the central law.

The analysis process is shown in Fig. 1.

### Survival analysis

The date of diagnosis and the date and cause of death were obtained from the Central Cancer Registry and treating clinicians. Median survival was estimated using the Kaplan–Meier method, and the difference was tested using the log‐rank test. We investigated the prognostic value of the selected genes in breast cancer patients using the ‘Kaplan–Meier plotter’ (KM plotter) database [21], in which updated gene expression data and survival information were obtained from 3955 breast cancer patients.

### Cell culture

The breast cancer cell line MCF‐7 was purchased from the Institute of Basic Medical Sciences, Chinese Academy of Medical Sciences (Beijing, China). And the human breast adenocarcinoma‐derived MCF‐7 cell line was maintained at 37 °C under 5% CO_2_ in Dulbecco’s modified Eagle’s/high glucose medium. The media were supplemented with 10% fetal bovine serum, 50 UI·mL^−1^ penicillin, and 50 μg·mL^−1^ streptomycin from HyClone (HyClone, Logan, UT, USA). Culture media were tested periodically and confirmed mycoplasma‐free.

### Cell viability assay

The detection time was 24 h after resveratrol treatment. Cell viability was determined using Cell Counting Kit‐8 (CCK‐8; Dojindo, Tokyo, Japan). Cells were seeded in 96‐well plates at a density of 0.2 × 10^4^ cells per well, and 10 μL of CCK‐8 reagent was added to each well at the indicated time points. The plates were incubated for 2 h at 37 °C, and the optical density (OD) of each well was measured at 450 nm for CCK‐8 according to the manufacturer’s instructions.

### Cell cycle analysis

Cells were collected via trypsin digestion and low‐speed centrifugation (×300 ***g***). The cells were washed once with PBS, fixed, permeabilized with prechilled 80% ethanol, and protected from light at −20 °C overnight. The ethanol was discarded by low‐speed centrifugation (×300 ***g***), and the cells were washed twice with stain buffer (BD Pharmingen^TM^, San Diego, CA, USA). Approximately 10^6^ cells per tube were stained with 500 μL PI/RNase staining buffer (BD Pharmingen^TM^) for 15 min at room temperature. The stain was washed from the sample using stain buffer, and the cells were resuspended in stain buffer. The samples were subjected to cell cycle distribution analysis in a BD FACSCelesta^TM^.

### Flow cytometry for apoptosis Analysis

Apoptosis of MCF‐7 cells was analyzed using flow cytometry. Cells were harvested 24 h after resveratrol treatment, washed with PBS, and resuspended in 100 μL of annexin kit buffer (BD Biosciences, San Jose, California, USA). The cells were stained with annexin‐V (BD Biosciences) and PI (BD Biosciences). Samples were placed in the dark for 15 min at room temperature, diluted in 400 μL of annexin kit buffer, and analyzed using BD FACSCelesta^TM^.

### RNA, miRNA isolation, and real‐time quantitative PCR (qPCR)

The experimental methods about total RNA isolation and qPCR of mRNA are similar to our previous experiments [9]. Total RNA was isolated using TriPure Isolation Reagent (Roche Diagnostics, Indianapolis, IN, USA). The DNA was removed from the sample using DNase I (Takara). The purity of the isolated RNA was determined using a Nanodrop‐2000 (Thermo) system. The A260/A280 ratio for each RNA sample was greater than 1.8, and the A260/A230 ratio was greater than 2.2. cDNA was synthesized by the following method. A total of 0.5‐2.0 μg of RNA was added to a 0.2‐mL PCR tube, and 1 μg of oligo (dT) was added. The final volume was increased to 12 μL with ddH_2_O. The temperature was rapidly reduced to 4 °C after heating at 70 °C for 5 min. Four microliters of 5 × M‐MLV buffer, 1 μL of M‐MLV reverse transcriptase, 1 μL of RNasin, and 1 μL of dNTPs were added, and the samples were incubated at 42 °C for 60 min followed by inactivation at 95 °C for 5 min. Reverse transcription reagents were purchased from Promega. MicroRNA was isolated using an EasyPure^®^ miRNA Kit (TransGen Biotech, Beijing, China). cDNA was synthesized using the miRcute Plus miRNA First‐Strand cDNA Kit (TIANGEN, Beijing, China).

Quantitative PCR was performed to examine the gene expression levels using the following primers from Genewiz (Suzhou, China). SYBR green dye was purchased from Takara. We used the miRcute Plus miRNA qPCR Kit (SYBR Green) (TIANGEN, Beijing, China) to examine the miRNAs. Amplification was performed with a polymerase activation step at 95 °C for 10 min and 40 cycles of 15 s at 94 °C and 30 s at 60 °C.

### Western blot

The method here regarding total protein isolation and western blot assay is similar to our previous experiments [9]. Equivalent amounts of protein (20 μg) that was extracted from the MCF‐7 cells with lysis buffer containing protease inhibitor cocktail were separated using gradient (4–12%) sodium dodecyl sulfate/polyacrylamide gel electrophoresis (SDS/PAGE), transferred onto a nitrocellulose filter membrane (Pall, BioTrace NT, USA), and blocked with 5% nonfat milk in TBST (10 mmol·L^−1^ Tris/HCl (pH 8.0), 150 mmol·L^−1^ NaCl, and 0.1% Tween‐20) for 2 h at room temperature. The membranes were washed 4 times with TBST buffer and incubated at 4 °C overnight with the appropriate primary rabbit antibodies. After washing four times with TBST, the immunoblots were incubated for 1 h at room temperature with a horseradish peroxidase‐labeled goat anti‐rabbit IgG secondary antibody. Each protein was detected in a chemiluminescence imaging analyzer (Tanon 5200, Shanghai, China) using Pierce ECL substrate (Thermo Scientific, Waltham, MA, USA).

The following antibodies were used in western blot detection: PARP (GeneTEX, Irvine, CA, USA; Cat. No. GTX100573), TP53 (Proteintech, Chicago, IL, USA; Cat. No. 10442‐1‐AP), CDK1 (GeneTEX, Irvine, CA, USA; Cat. No. GTX108120), BCL2 (Proteintech, Chicago, IL, USA; Cat. No. 12789‐1‐AP), BAX (Proteintech, Chicago, IL, USA; Cat. No. 50599‐2‐Ig), and GAPDH (GeneTEX, Irvine, CA, USA; Cat. No. GTX100118).

### Statistical analysis

Experiments were performed at least three times with similar results. Statistical comparisons were made using one‐way analysis of variance (ANOVA) after ascertaining the homogeneity of variance between the different treatments. The analysis of different times and treatments was performed using t tests, and *P* < 0.05 was considered significant. Statistical analyses were performed using R (v4.0.2).

### Primer design

The primer list used for validation is presented in the Supplement File (Tables S1 and S2). Primer pairs and probes were designed using Primer3 software according to the operating manual, and their specificity was determined using the Primer‐blast program.

### Ethics statement

All clinical data involved in this study were obtained from open databases and therefore met ethical review standards.

## Results

### Data mining reveals that resveratrol inhibits the proliferation of MCF‐7 cells

**Fig. 2:**
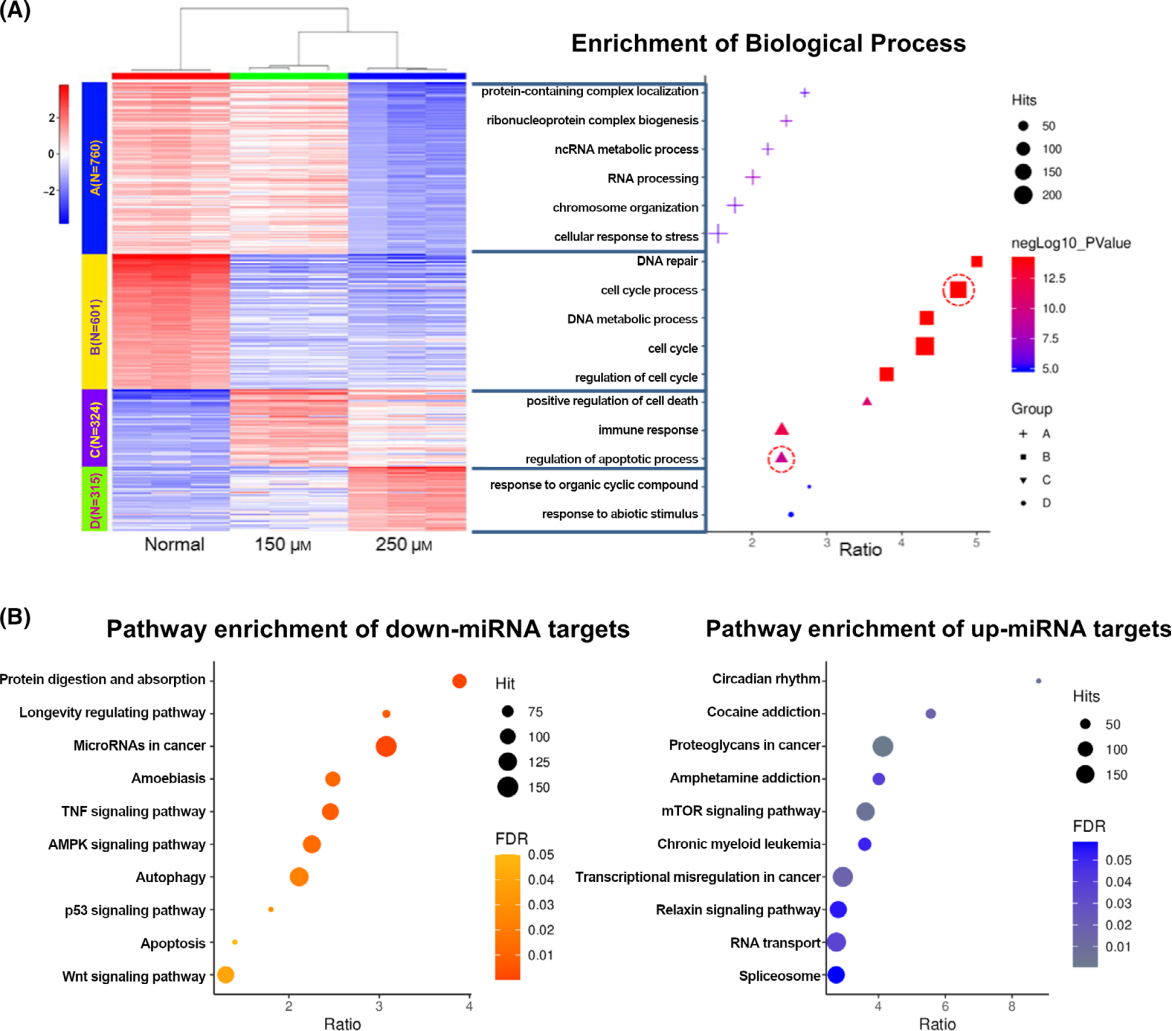
Enrichment analysis of mRNAs and miRNAs in MCF‐7 cells regulated by resveratrol. (A) GO enrichment analysis for each of the 4k‐means clusters to obtain the major biological processes. (B) KEGG pathway enrichment results for target genes of downregulated expressed miRNAs. (C) KEGG pathway enrichment results for target genes of upregulated expressed miRNAs.

To reveal the molecular pathway of resveratrol‐induced inhibition of MCF‐7 cell proliferation, we performed a secondary analysis of the gene expression profile dataset (GSE25412). The integration of K‐means clustering and biological process enrichment showed that the downregulation of the cell cycle and activation of apoptosis were primarily responsible for the inhibition of cell proliferation, which echoed a previous experiment [22] (Fig. 2A). The results of the miRNA target enrichment showed that the main target genes of downregulated miRNAs were the TNF signaling pathway, autophagy, and apoptosis, and the main pathway of enrichment of upregulated target gene miRNAs was amphetamine addiction and spliceosome. These results suggest that resveratrol plays a role in regulating mRNA via miRNAs (Fig. 2B). These results revealed changes in gene expression profiles indirectly affected by resveratrol via miRNAs.

### Resveratrol inhibits the proliferation of MCF‐7 cells by blocking the cell cycle and promoting apoptosis

**Fig. 3:**
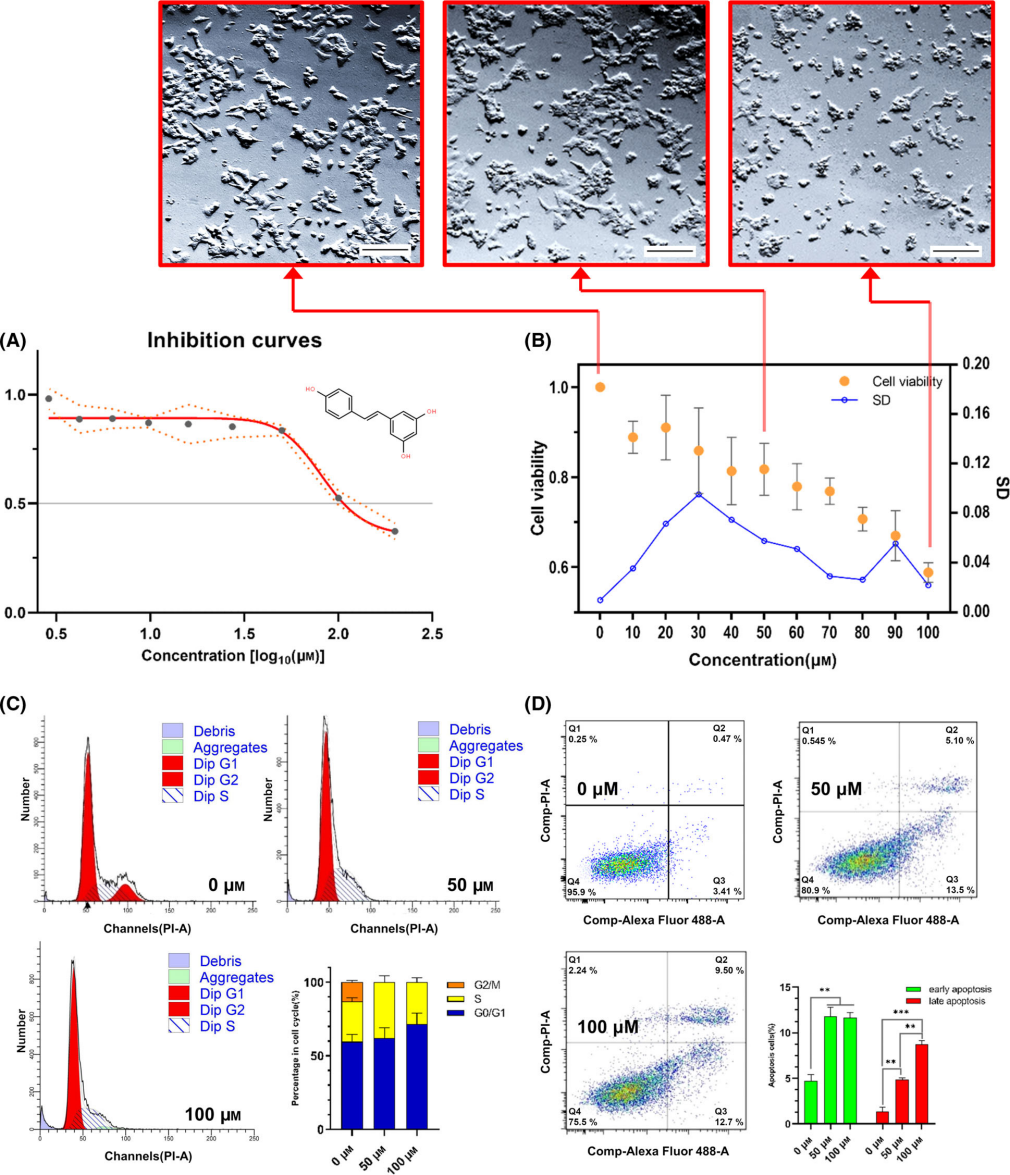
Resveratrol inhibited the proliferation of MCF‐7 cells. (A) Growth inhibition rate curve of resveratrol in MCF‐7 cells. (B) The viability of MCF‐7 cells decreased gradually with increasing resveratrol treatment concentration. The micrograph magnification was 100× (scale bar = 200 μm). (A, B) Data represented mean  ±  SD of *n* = 6 independent experiments. ANOVA was used for statistical analysis. (C) Resveratrol treatment inhibited the MCF‐7 cell cycle, resulting in the disappearance of the G2/M phase. (D) Resveratrol treatment promoted MCF‐7 apoptosis. Data represented mean  ± SEM of *n* = 3 independent experiments. A two‐tailed Student's t‐test was used for statistical analysis. ** means *P* < 0.01; *** means *P* < 0.001.

To further verify the inhibitory effect of resveratrol on MCF‐7 cell proliferation, we tested different doses of resveratrol for 24 h. The inhibition curve showed an IC_50_ of 107.32 μm (Fig. 3A). We observed changes in variance and found that the dose response of MCF‐7 cells to resveratrol tended to stabilize at 40 μm (Fig. 3B). Therefore, we chose concentrations of 50 μm and 100 μm for the next study. Flow cytometry analysis further showed that 50 μm and 100 μm resveratrol arrested MCF‐7 cells in S phase (Fig. 3C and Table S3). The inhibition of resveratrol on the cell cycle was gradually enhanced with increasing treatment concentrations (Fig. S1). Compared to 50 μm resveratrol, the apoptosis rate of MCF‐7 cells treated with 100 μm resveratrol was higher (Fig. 3D).

### Prediction of the upstream signal process of resveratrol

**Fig. 4:**
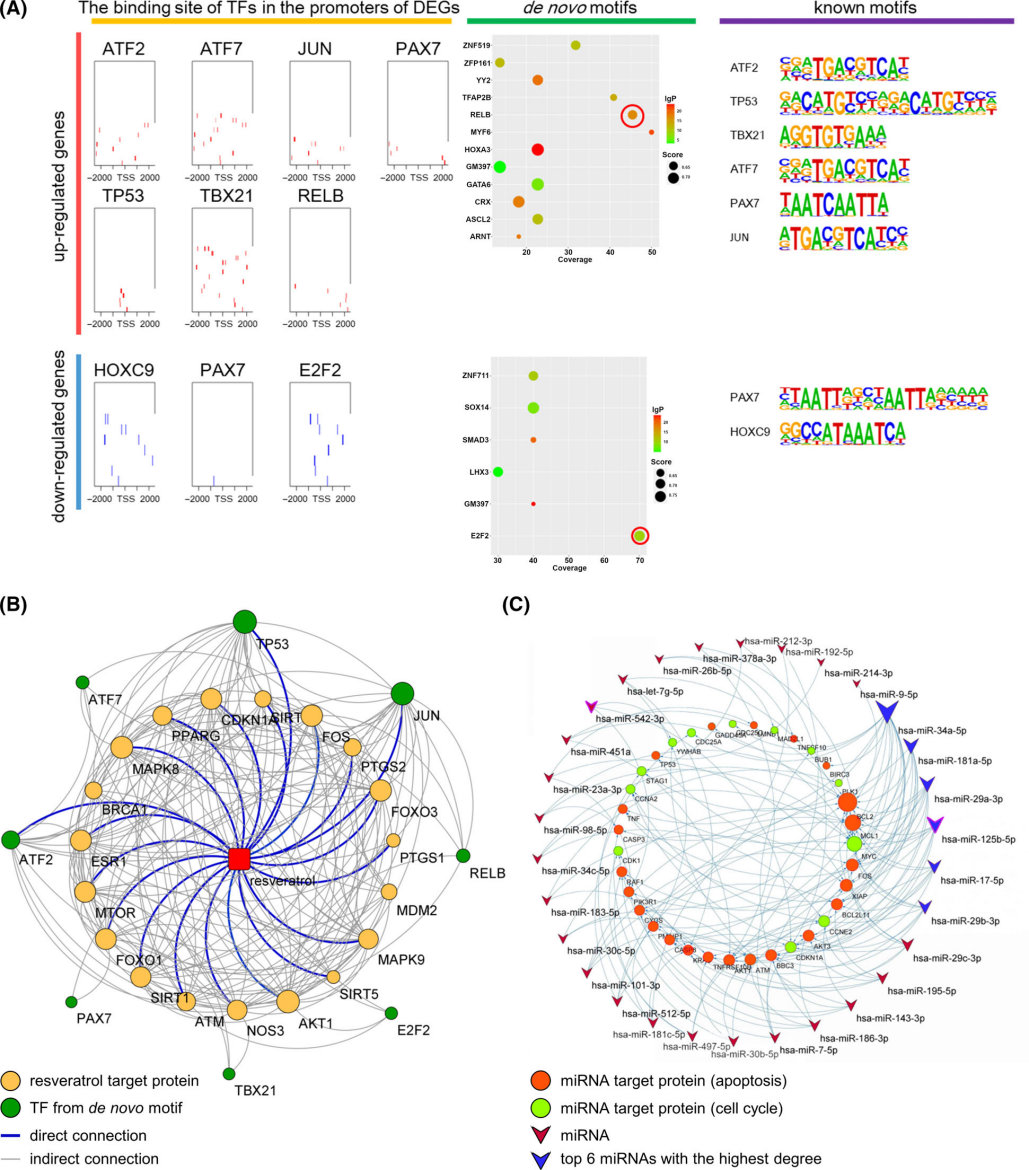
Resveratrol target protein prediction and miRNA regulatory network. (A) The motifs of DEGs were obtained from Homer. (B) Prediction of the interaction network of target proteins and transcription factors of resveratrol in MCF‐7 cells. [Red: resveratrol; yellow: target proteins; green: motif‐binding transcription factor; gray line: indirect connection between TF and resveratrol; blue line: directly related to TF and resveratrol] (C) MicroRNA‐mRNA network. [Red circle: apoptosis pathway; green circle: cell cycle; purple polygon: miRNA; blue polygon: miRNA with the highest degree. The shape size represented the degree of connectivity].

To explore the transcription pattern of resveratrol effects on the cell cycle and apoptosis of MCF‐7 cells, we analyzed the DEGs in the above pathways using *de novo* motif analysis. The results showed that when the screening threshold score was higher than 0.6 and covered more than 10% of the target sequence, RELB and E2F2 had higher coverage and confidence (Fig. 4A). Eight known transcription factor motifs were found in the promoters of the upregulated genes (Fig. 4A), and two motifs were identified in the promoters of the downregulated gene (Fig. 4A). The interaction network diagram indicated that resveratrol directly regulated 3 transcription factors and 17 target genes and directly or indirectly affected the motifs (Fig. 4B). A previous analysis found that downregulated miRNAs may be responsible for the main post‐transcriptional regulation of the activation of apoptotic genes. We chose the top 30 miRNAs with the highest target number of predicted key hub genes of the cell cycle and apoptosis. We found that miRNA downregulation increased the expression of apoptotic genes, and the top 6 miRNAs (hsa‐miR‐34a‐5p, hsa‐miR‐181a‐5p, hsa‐miR‐29a‐3p, hsa‐miR‐125b‐5p, hsa‐miR‐17‐5p, and hsa‐miR‐29b‐3p) with node connectivity greater than 6 were selected for subsequent experimental validation (Fig. 4C).

### Resveratrol affects gene expression patterns of the cell cycle and apoptosis

**Fig. 5:**
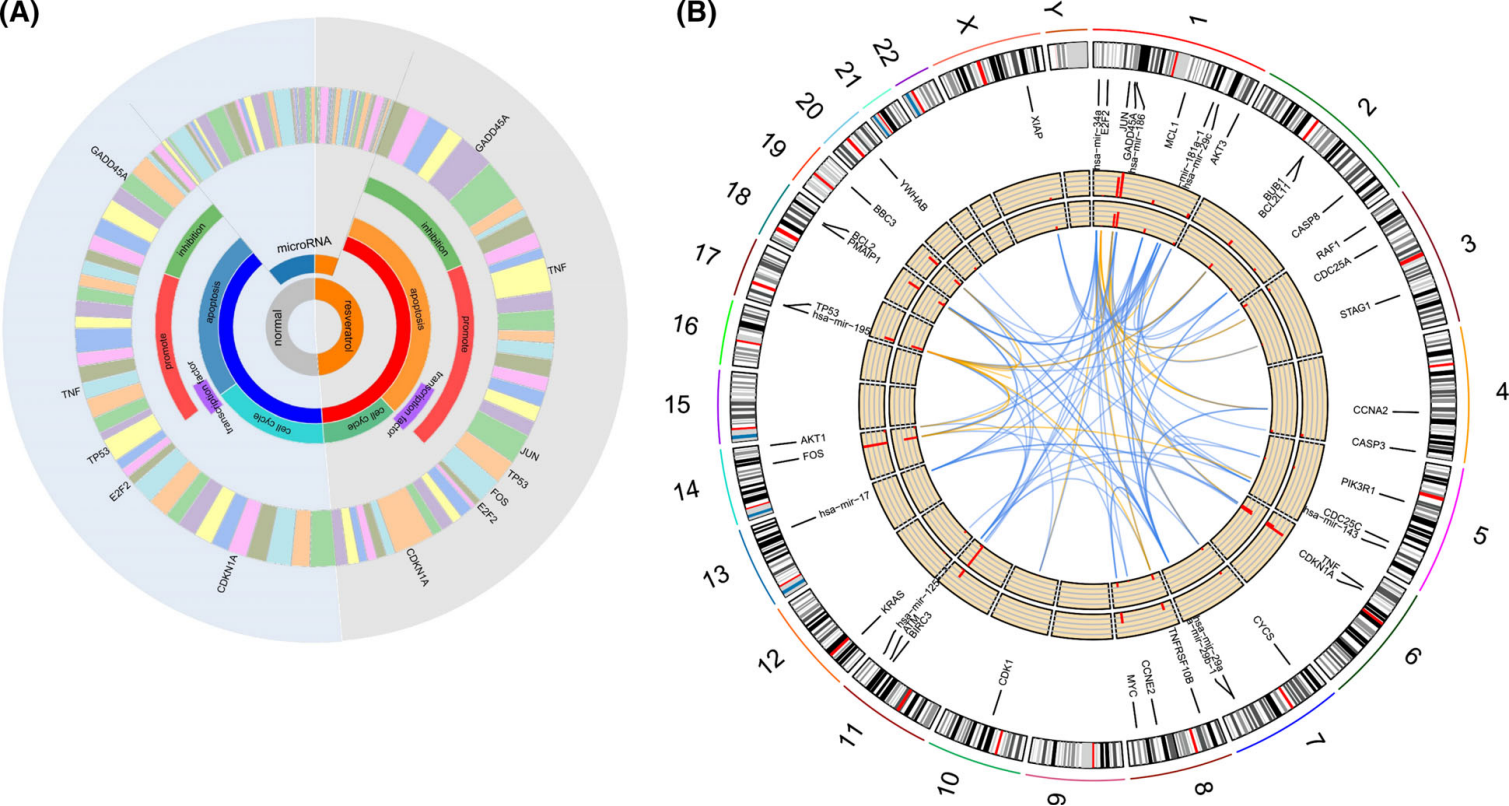
Resveratrol regulated gene expression of the cell cycle and apoptosis. (A) Resveratrol regulated the expression of miRNAs and promotes apoptosis. The graph showed the quantitative comparison results of the total amount of RNA (miRNA‐mRNA network) treated with or without resveratrol. The ring area was classified according to the proportion of the same floor. The layers from the inside to the outside were groups of miRNAs, pathways (mRNAs), transcription factors, effects, and specific genes corresponding to the outermost layer. Resveratrol treatment reduced miRNA and increased apoptosis, and pro‐apoptotic transcription factors increased significantly. Although the proportion of apoptotic cells that were inhibited also increased, it contained some DNA damage repair proteins, such as GADD45A, which inhibited the cell cycle. (B) Genomic mapping of the top 10 miRNAs, cell cycle and apoptosis genes. The outer circle histogram represented the fold change in expression after 250 μm resveratrol treatment; the inner circle histogram represents the fold change in expression after 150 μm resveratrol treatment. The blue line in the center indicated the miRNA targeting relationship, and the orange line indicated the transcription regulation relationship.

Resveratrol treatment generally reduced the level of gene transcription related to the cell cycle and apoptosis pathways in MCF‐7 cells, and we found that the downregulation of miRNA upregulated the apoptosis pathway (Fig. 5A). Mapping of the miRNAs and selected genes to the genome clearly revealed the combined effects of miRNA and transcription factor regulation on changes in gene expression. For example, miRNAs (hsa‐miR‐125b targets TP53; hsa‐miR‐195 and hsa‐miR‐186 target CDK1) and transcription factors (TP53 and FOS transcriptional regulation TP53; E2F2 transcriptional regulation CDK1) regulated the expression changes in TP53 and CDK1 (Fig. 5B).

### Prognosis Analysis and expression‐level validation of hub molecules

**Fig. 6:**
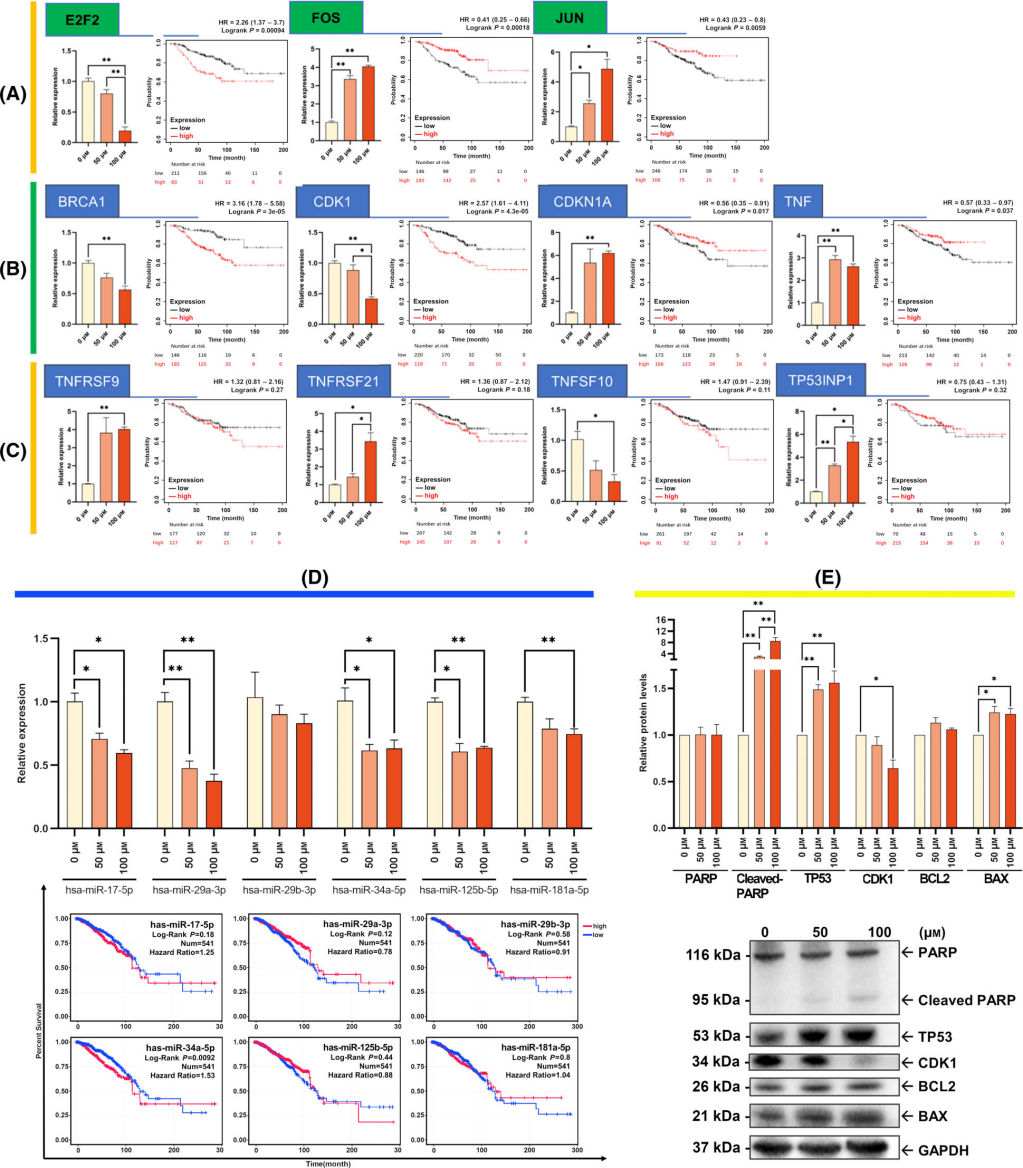
Quantitative detection and survival analysis of key DEGs and miRNAs. (A) qPCR was used to detect the mRNA levels of the three transcription factors and their survival curves. Green labels indicated TFs. (B) qPCR detection significantly improved the survival rate of gene mRNA levels. (C) The mRNA expression levels of genes that were differentially expressed but did not significantly affect prognosis. (D) qPCR detection and survival analysis of hub miRNAs. (E) Western blot analysis of the key factors of cell cycle arrest and apoptosis. Data represented mean  ± SEM of *n* = 3 independent experiments. A two‐tailed Student's t‐test was used for statistical analysis. * means *P* < 0.05; ** means *P* < 0.01.

To further detect the roles of resveratrol target DEGs in prognosis, we performed survival analysis of key hub molecules and validated the reliability of their RNA expression levels using qPCR. Our results showed that the downregulation of transcription factors (E2F2, JUN, and FOS) significantly improved patient survival (Fig. 6A). Among the hub genes, the downregulation of BRCA1, CDK1 CDKN1A, and TNF produced a significant improvement in patient survival, but the downregulation of TNFRSF9, TNFSF10, TNFRSF21, and TP53INP1 did not induce a better prognosis (Fig. 6B,C). Only the downregulation of hsa‐miR‐34a‐5p was associated with a significant improvement in patient survival (Fig. 6D). The qPCR results of the above RNAs showed trends consistent with the expression profiles. We selected 5 representative genes (PARP, TP53, CDK1, BCL2, and BAX) at the protein level for further validation using western blotting, which confirmed cell cycle arrest and apoptosis activation (Fig. 6E). The protein levels further suggested that resveratrol inhibited MCF‐7 cell proliferation via the cell cycle and apoptosis.

### Resveratrol relies on TP53 to regulate the cell state in MCF‐7 cells

**Fig. 7:**
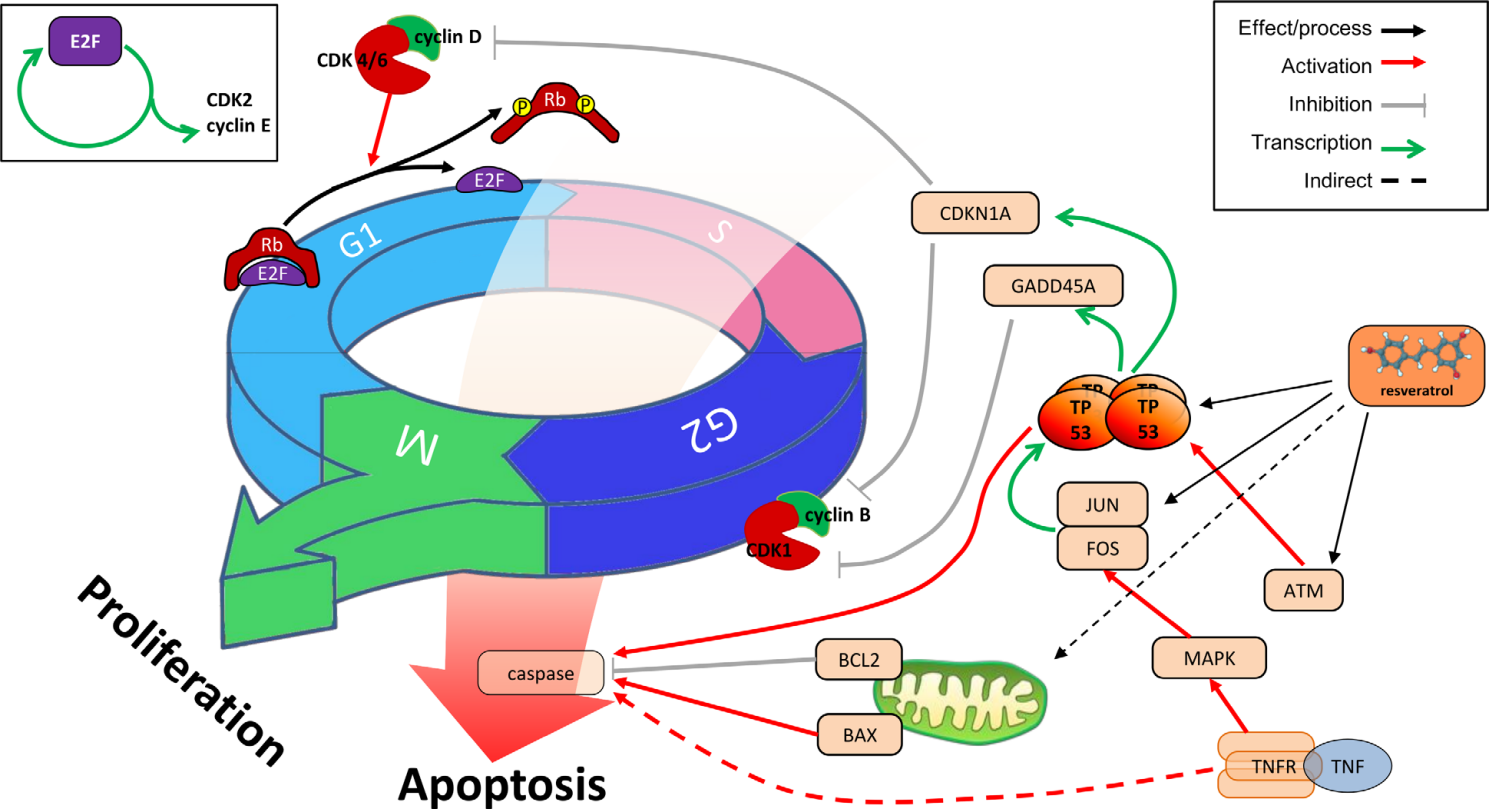
Resveratrol regulation on the proliferation of MCF‐7 cells. The inhibition of resveratrol on MCF‐7 cell proliferation involved many factors, including classical cell cycle and apoptosis. Connecting these factors revealed that TP53 protein was an important hub in the process.

These results suggested the regulatory effect of resveratrol on the proliferation of MCF‐7 cells. Resveratrol inhibited cell proliferation by regulating the expression of some genes, and TP53 is the core in cell cycle and apoptosis (Fig. 7). Resveratrol seemed to play a role in the post‐transcription regulation of miRNAs, but the transcriptional expression pattern of miRNAs was not very clear.

## Discussion

The inhibitory effects and ‘‐omic’ data on resveratrol are widely published. Based on improved data analysis methods and functional gene enrichment databases, the present study reconstructed a transcriptional regulatory model of multimolecular interactions of resveratrol in inhibiting breast cancer cell (MCF‐7) proliferation using a combination of secondary mining of published data and experimental validation.

Analysis of DEG pathway enrichment and the STITCH network found that resveratrol inhibited the proliferation of MCF‐7 cells primarily via the response of node genes of the cell cycle and apoptosis. Due to the development of detection technology and the openness of data, we integrated the upstream and downstream signal networks of resveratrol in MCF‐7 cells via *de novo* motifs. First, we predicted the target protein of resveratrol and found a role for many different biological processes. We combined the results of the upstream *de novo* motif of the cell cycle and apoptosis and DEGs and found that the mechanism of resveratrol involved blockade of the cell cycle and induction of apoptosis via the upregulation (CDKN1A, JUN, FOS, and TP53INP1) and downregulation (BRCA1, CDK1, and E2F2) of a set of genes on MCF‐7 cells. JUN and TP53 had the highest weight of the upregulated transcription factors, and E2F2 had the highest coverage in the downregulated expression. JUN plays a broad role in cell differentiation, proliferation, and apoptosis [23]. TP53 also regulates apoptosis, but the relationship between the two genes in cell apoptosis depends on the cell type [23]. The mutation status of the TP53 gene in tumors directly affects its transcriptional regulation [24]. MCF‐7 cells, which have a wild‐type TP53 gene, were used as a research model [25]. The present study found that resveratrol upregulated the transcription levels of JUN and FOS, but TP53 did not show differences in transcriptional expression in the data analysis. Although TP53 was not a differentially significant gene at the RNA level, it was significantly upregulated at the protein level, which suggests that TP53 plays a dominant role in the interaction network via post‐transcriptional modification. E2F2 is a member of the E2F transcription factor family, and it plays a vital role in cell cycle progression. Previous studies showed that the destruction of the E2F2 gene decreased the S phase entry rate [26], which confirmed that Rb‐E2F was a restriction point for cells in the G1 phase to enter S phase in the cell cycle [27, 28, 29]. We found that resveratrol treatment of MCF‐7 cells decreased the transcription level of the E2F family, and E2F2 played a related role between resveratrol and the cell cycle (Table S4, Fig. 5A). Therefore, resveratrol acted on MCF‐7 cells to cause cycle arrest and apoptosis, primarily via the regulation of JUN, TP53, and E2F2.

Second, miRNAs are a method of post‐transcriptional gene expression regulation. Resveratrol may also play a regulatory role by targeting miRNAs that target the cell cycle and apoptosis. Cloonan et al. demonstrated that hsa‐miR‐7‐5p promoted cell growth and increased the S phase by targeting CDKN1A (P21) [30]. When miR‐17 expression decreased, we also detected an upregulation of CDKN1A. The miR‐29 family primarily promotes the tissue invasion and metastasis of tumor cells while targeting BAX to prevent apoptosis [31, 32]. When resveratrol reduced hsa‐miR‐29a‐3p, BAX was increased and may promote cell apoptosis. Hsa‐miR‐34a‐5p is highly expressed in tumor tissues and is closely related to the survival rate [33]. Hsa‐miR‐34a‐5p also acts on the cell cycle and induces apoptosis when it is knocked out [34]. We found that resveratrol reduced the expression of hsa‐miR‐34a‐5p, which helped improve patient survival. Hsa‐miR‐125b‐5p inhibits apoptosis by targeting DRAM or TRIAP1 [35, 36]. Wei et al. confirmed that hsa‐miR‐181a‐5p inhibited the apoptosis and autophagy of MCF‐7 cells [37]. We determined that the increase in downregulated miRNAs and apoptosis was consistent with existing reports by detecting apoptosis and the apoptotic marker protein PARP. By investigating the expression of miRNAs and their target genes, we noted that the expression of 6 miRNAs, hsa‐miR‐17‐5p, hsa‐miR‐29a‐3p, hsa‐miR‐29b‐3p, hsa‐miR‐34a‐5p, hsa‐miR‐125b‐5p, and hsa‐miR‐181a‐5p, negatively correlated with their target genes. This change also contributed to the increased expression of the high degree genes in the miRNA‐mRNA network.

For the cell cycle, resveratrol acted on proteins such as JUN, FOS, ATM, and TP53, which affected the expression of proteins, such as GADD45A and CDKN1A. ATM and ATR share responsibilities as apical protein kinases in all known cell cycle checkpoints in mammalian cells, with the possible exception of the mitotic spindle checkpoint, which is activated by treatment with an inhibitor of microtubule polymerization, nocodazole [38]. GADD45A and CDKN1A are effector genes of TP53. GADD45A interacts with CDK1, and its inhibitory effect on the cell cycle (G2 phase) is achieved by destroying the CDK1‐cyclin B1 complex [39, 40]. The cell cycle results confirmed this process. Compared with GADD45A, CDKN1A has a broader inhibitory effect on the CDK family [41]. Further breakdown of the cell cycle test results suggested a differential sensitivity of these two proteins to resveratrol (Fig. S1). The effect of resveratrol on apoptosis involves mitochondria, TNF and TP53. DNA damage triggers TP53‐mediated cell apoptosis [42]. BAX characterizes the occurrence of the mitochondrial apoptotic pathway [43]. TNF‐TNFR1 is one of two important models of the external stage of apoptosis [44]. The boundaries between these three processes are not obvious. However, it has limited cleavage activity for PARP because caspase 3 of MCF‐7 is a mutant type [45]. Our results showed that TP53, BAX, and TNF were upregulated, and PARP was cleaved after resveratrol treatment. Combined with the *de novo* motif results, we revealed that there were two positive feedback regulation loops (E2F and TP53) in the cycle arrest and the promotion of apoptosis caused by resveratrol treatment in MCF‐7 cells, and they may be the core factors of resveratrol.

We also tested the effects of resveratrol on other wild‐type TP53 cell lines by performing the above experiment in the ZR‐75‐1 cell line in vitro. The results showed that resveratrol treatment did not significantly affect TP53 protein levels in ZR‐75‐1 cells (Fig. S2D), but cell proliferation was inhibited, and cell cycle and apoptosis showed similar results as the MCF‐7 cells (Fig. S2A‐C). This result may be attributed to the inhibitory effect of resveratrol on HER2 [46]. These results suggest that resveratrol inhibits proliferation in other wild‐type TP53 cell lines, but its molecular effects in different cell types are not identical. This result highlights the importance of molecular diagnosis in the treatment of breast cancer.

## Conclusions

In conclusion, we demonstrated that resveratrol inhibited the proliferative viability of MCF‐7 cells by regulating the expression of TF and miRNA and acting on genes affecting the cell cycle and apoptosis, with TP53 and E2F as the dominant hub genes. Survival analysis revealed that most of the target genes regulated by resveratrol significantly improved prognosis.

## Conflict of interest

The authors declare no conflict of interest.

## Author contributions

ZL, HY, JZ, and YZ involved in conception and design. ZL and JZ performed administrative support. JZ, WL, and DZ involved in provision of study materials or patients. YZ, ZW, YZ, and WL performed collection and assembly of data. YZ, HY, and ZL analyzed and interpreted the data. All authors wrote and finally approved the manuscript.

## Supporting information


**Fig. S1.** Flow cytometry cell cycle detection.
**Fig. S2.** Generalization tests of the effect of resveratrol on another wild‐type TP53 breast cancer cell (ZR‐75‐1).
**Table S1.** List of qPCR primers for miRNA.
**Table S2.** List of qPCR primers for mRNA.
**Table S3.** The expression level of some histones from GSE25412.
**Table S4.** The expression level of E2F family from GSE25412.

## Data Availability

The GSE25412 dataset was derived from a public database, and the PCR array expression data of miRNAs were taken from the research report of Venkatadri et al. (PMID: 26890143; Copyright and License information: http://creativecommons.org/licenses/by/4.0/).
