# Supplementary material for: Integrative investigation of the TF–miRNA coregulatory network involved in the inhibition of breast cancer cell proliferation by resveratrol
Source: FEBS Open Bio. 2021 Dec 12;12(2):379–93. doi: 10.1002/2211-5463.13344 (PMC8804603; doi:10.1002/2211-5463.13344)
Supplement: Supplementary file 1 — Fig. S1. Flow cytometry cell cycle detection. Fig. S2. Generalization tests of the effect of resveratrol on another wild‐type TP53 breast cancer cell (ZR‐75‐1). Table S1. List of qPCR primers for miRNA. Table S2. List of qPCR primers for mRNA. Table S3. The expression level of some histones from GSE25412. Table S4. The expression level of E2F family from GSE25412. [file FEB4-12-379-s001.docx]

Supplementary Material

Catalog

[Figure S1| Flow cytometry cell cycle detection. 2](#_Toc84146331)

[Figure S2| Generalization tests of the effect of resveratrol on another wild type TP53 breast cancer cell (ZR-75-1). .......................................................................................................................................................3](#_Toc84146332)

[Table S1| List of qPCR primers for miRNA. 4](#_Toc84146333)

[Table S2| List of qPCR primers for mRNA. 5](#_Toc84146334)

[Table S3| The expression level of some histones from GSE25412 6](#_Toc84146335)

[Table S4| The expression level of E2F family from GSE25412 7](#_Toc84146336)


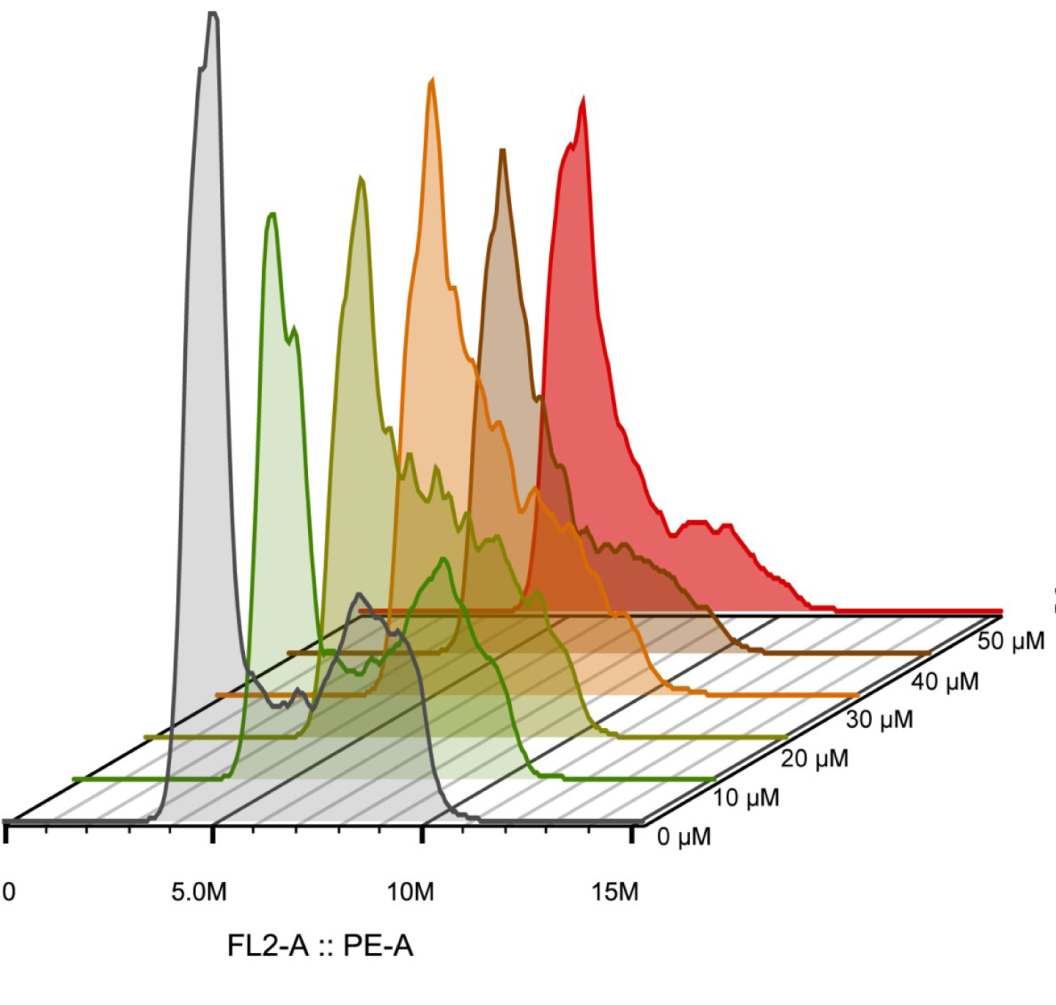


# Figure S1| Flow cytometry cell cycle detection.

The PI channel peak of flow cytometry gradually changed as the concentration of resveratrol-treated MCF-7 cells increased. This result indicated that the inhibitory effect of resveratrol on cell cycle shifted from initial G2 phase inhibition to S phase inhibition with increasing treatment concentration. The G0/G1 phase peaks showed at 0.5 M; the G2/M phase peaks showed at 1.0 M.


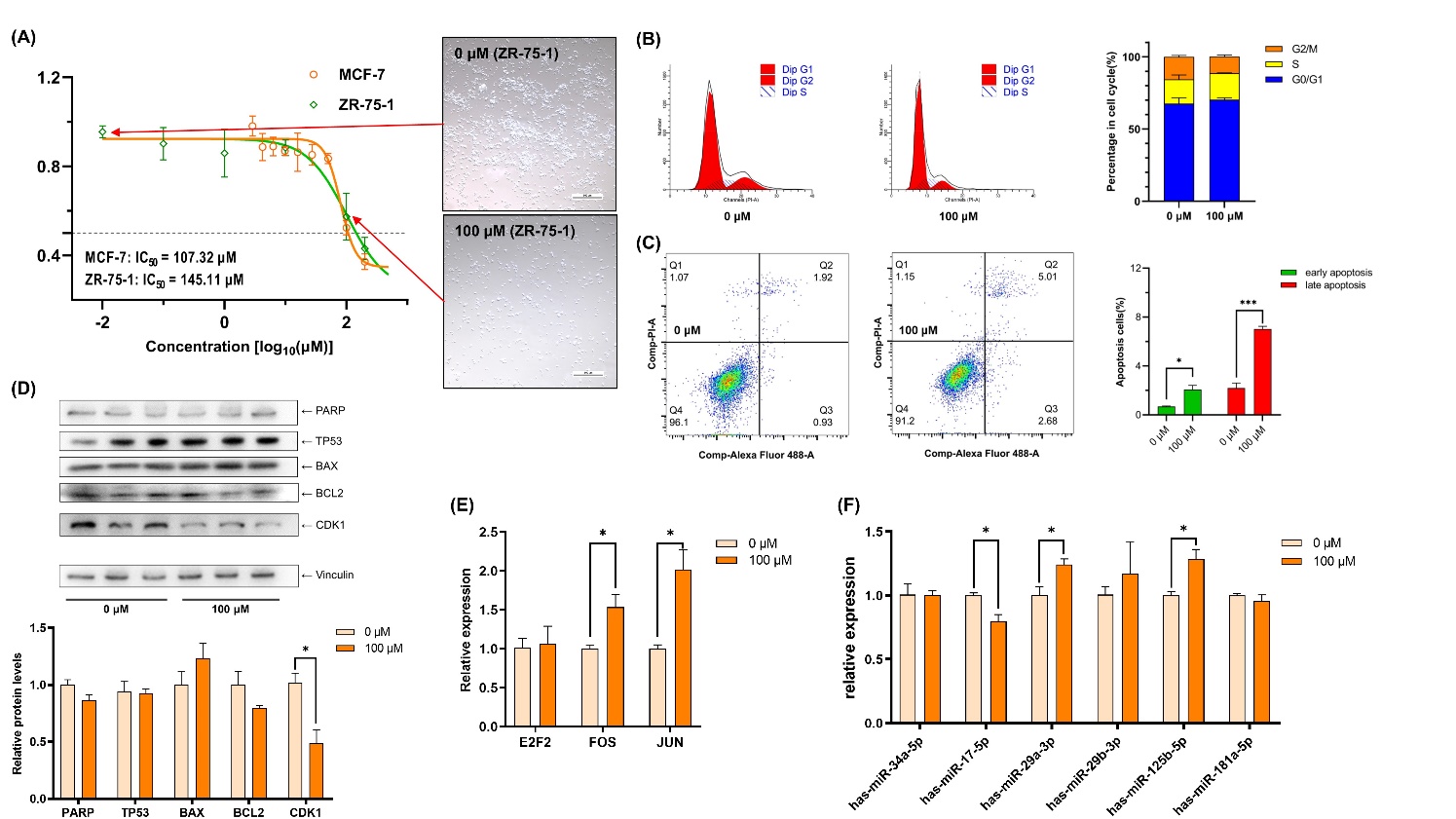


# Figure S2| Generalization tests of the effect of resveratrol on another wild-type TP53 breast cancer cell (ZR-75-1).

(A) The inhibition rate curve of resveratrol on ZR-75-1 was flatter compared to that on MCF-7. Similarly, we selected treatment concentrations (100 μM) with nearly half inhibition rate for subsequent assays. (B) The results of the cell cycle assay showed that resveratrol treatment inhibited the G2/M phase of ZR-75-1 cells. (C) Flow cytometry for apoptosis assay revealed that resveratrol significantly increased the apoptosis rate of lZR-75-1. (D) Western blot assay found that resveratrol treatment caused a significant decrease in CDK1, an increase in BAX and a decrease in BCL2 in ZR-75-1. (E) The results of qPCR show that resveratrol treatment increased JUN and FOS transcript levels in ZR-75-1. (F) Expression of hsa-miR17-5p was down-regulated and expression of hsa-miR29a-3p and hsa-miR125b-bp was up-regulated in 6 miRNAs quantified after resveratrol treatment of ZR-75-1 cells. “*” means p < 0.05; “**” means p < 0.01; “***” means p < 0.001.

# Table S1| List of qPCR primers for miRNA.

| Name | Sequence (5’->3’) |
| --- | --- |
| hsa-miR-34a-5p | ACACTCCAGCTGGGTTGGCAGTGTCTTAG |
| hsa-miR-29a-3p | ACACTCCAGCTGGGTTAGCACCATCTGAA |
| hsa-miR-125b-5p | ACACTCCAGCTGGGTTCCCTGAGACCCTA |
| hsa-miR-181a-5p | ACACTCCAGCTGGGTAACATTCAACGCTGT |
| hsa-miR-29b-3p | ACACTCCAGCTGGGTTAGCACCATTTGAAA |
| hsa-miR-17-5p | ACACTCCAGCTGGGTCAAAGTGCTTACAGT |

† The kit already contains universal downstream primers.

# Table S2| List of qPCR primers for mRNA.

| Gene symbol | Forward (5’->3’) | Reverse (5’->3’) |
| --- | --- | --- |
| BRCA1 | AAGCGAGCAAGAGAATCC | TGTACCATCCATTCCAGTTG |
| CDKN1A | GATTAGCAGCGGAACAAG | GCCAGGAAAGACAACTAC |
| CDK1 | TGCTTATGCAGGATTCCAGGT | GAATCCATGTACTGACCAGGAGG |
| E2F2 | GCCCAGCTACTGCTACCTAC | GTTGGGAACTCAGGGACGAC |
| JUN | GTGCCAACTCATGCTAAC | GTTCTCAAGTCTGTCTCTCT |
| FOS | CAGACTACGAGGCGTCATCC | CGTGGGAATGAAGTTGGCAC |
| TP53INP1 | AGCTGGTTTATCACCCCACC | CCAGGGCAGGAGTTATGCAC |
| TNF | CATCTGGAATCTGGAGAC | CTGGAAACATCTGGAGAG |
| TNFRSF9 | GCTCTTCCTGCTGTTCTT | AGTTCACATCCTCCTTCTTC |
| TNFSF10 | CAGAGGAAGAAGCAACAC | GGATGACCAGTTCACCAT |
| TNFRSF21 | GTGCCTTCTAGTGTGATGA | GTTGTCTGTCTCCTTGGT |
| GAPDH^*^ | CCATGAGAAGTATGACAACAG | GTCCTTCCACGATACCAA |
| U6^*^ | CTCGCTTCGGCAGCACA | AACGCTTCACGAATTTGCGT |

† Internal reference genes.

# Table S3| The expression level of some histones from GSE25412

| Gene symbol | Group (VALUE) | | |
| --- | --- | --- | --- |
|  | 0 μM | 150 μM | 250 μM |
| HIST2H2AC | 11.29 | 9.61 | 8.45 |
| HIST2H2AB | 11.98 | 9.39 | 8.77 |
| HIST1H2BK | 10.71 | 8.32 | 8.81 |
| HIST1H4C | 9.91 | 7.75 | 8.23 |
| HIST1H1E | 11.50 | 9.39 | 9.88 |
| HIST1H2BF | 8.41 | 5.43 | 5.87 |
| HIST1H2BH | 9.58 | 7.25 | 7.62 |
| HIST1H2BI | 6.30 | 4.05 | 4.83 |
| HIST1H2BM | 10.58 | 5.43 | 6.73 |
| HIST1H3B | 8.10 | 5.09 | 6.00 |
| HIST1H2AB | 8.33 | 4.72 | 6.09 |
| HIST1H2BC | 6.82 | 4.78 | 4.81 |
| HIST1H4D | 8.12 | 6.07 | 8.17 |
| HIST1H3D | 8.94 | 6.79 | 7.66 |
| HIST1H1D | 7.34 | 5.13 | 5.90 |
| HIST1H3F | 8.85 | 6.19 | 6.61 |
| HIST1H3G | 7.89 | 4.82 | 5.89 |
| HIST1H2AJ | 6.21 | 4.14 | 5.39 |
| HIST1H1B | 9.82 | 6.00 | 6.88 |
| HIST1H3I | 10.95 | 8.34 | 8.18 |
| HIST1H3J | 7.84 | 5.42 | 7.14 |

The reduced expression of histones also confirmed resveratrol-induced S phase inhibition in another way.

# Table S4| The expression level of E2F family from GSE25412

| Gene symbol | Group (VALUE) | | |
| --- | --- | --- | --- |
|  | 0 μM | 150 μM | 250 μM |
| E2F1 | 7.80 | 7.33 | 7.18 |
| E2F2 | 6.78 | 5.20 | 5.17 |
| E2F3 | 6.40 | 5.85 | 5.04 |
| E2F4 | 9.28 | 8.79 | 8.86 |
| E2F5 | 6.85 | 6.71 | 5.42 |
| E2F6 | 9.14 | 8.76 | 8.58 |
| E2F7 | 7.72 | 7.56 | 7.62 |
| E2F8 | 7.20 | 4.71 | 4.38 |
